# An mRNA vaccine encoding the SARS-CoV-2 receptor-binding domain protects mice from various Omicron variants

**DOI:** 10.1038/s41541-023-00800-0

**Published:** 2024-01-02

**Authors:** Ryuta Uraki, Masaki Imai, Mutsumi Ito, Seiya Yamayoshi, Maki Kiso, Nao Jounai, Kazuki Miyaji, Kiyoko Iwatsuki-Horimoto, Fumihiko Takeshita, Yoshihiro Kawaoka

**Affiliations:** 1 Division of Virology, Institute of Medical Science, The University of Tokyo, Tokyo, 108-8639 Japan; 2 The Research Center for Global Viral Diseases, National Center for Global Health and Medicine Research Institute, Tokyo, 162-8655 Japan; 3 Biologics Division, Vaccine Research Laboratories, Daiichi Sankyo Co., Ltd, Tokyo, 134-0081 Japan; 4 Influenza Research Institute, Department of Pathobiological Sciences, School of Veterinary Medicine, University of Wisconsin-, Madison, WI 53711 USA; 5 The University of Tokyo, Pandemic Preparedness, Infection and Advanced Research Center, Tokyo, 162-8655 Japan

**Keywords:** RNA vaccines, RNA vaccines, SARS-CoV-2

## Abstract

Here, we assessed the efficacy of a lipid nanoparticle-based mRNA vaccine candidate encoding the receptor-binding domain (LNP-mRNA-RBD) in mice. Mice immunized with LNP-mRNA-RBD based on the ancestral strain (ancestral-type LNP-mRNA-RBD) showed similar cellular responses against the ancestral strain and BA.5, but their neutralizing activity against BA.5 was lower than that against the ancestral strain. The ancestral-type LNP-mRNA-RBD protected mice from the ancestral strain or BA.5 challenge; however, its ability to reduce the viral burdens after BA.5 challenge was limited. In contrast, immunization with bivalent LNP-mRNA-RBD consisting of the ancestral-type and BA.4/5-type LNP-mRNA-RBD or monovalent BA.4/5-type LNP-mRNA-RBD elicited robust cellular responses, as well as high and moderate neutralizing titers against BA.5 and XBB.1.5, respectively. Furthermore, the vaccines containing BA.4/5-type LNP-mRNA-RBD remarkably reduced the viral burdens following BA.5 or XBB.1.5 challenge. Overall, our findings suggest that LNP-mRNA-RBD is effective against SARS-CoV-2 infection.

## Introduction

The ongoing global pandemic caused by severe acute respiratory syndrome coronavirus 2 (SARS-CoV-2) has led to worldwide public health and economic damages. The World Health Organization (WHO) reported that, as of May 2023, there have been over 760 million cases of coronavirus disease 2019 (COVID-19), including 6.9 million deaths.

Vaccination remains the most-effective first line of defense against infectious pathogens. Several modalities have been used to develop COVID-19 vaccines targeting the spike (S) protein of SARS-CoV-2 (e.g., lipid nanoparticle-encapsulated mRNA, viral-vector-based vaccines, inactivated virions), which allowed their rapid and global implementation (https://covid19.who.int). These vaccines have been effective in reducing the rates of infection, hospitalizations, and mortality. However, continuous efforts are required to enhance immune responses to vaccination and develop alternative vaccine strategies to control current and future SARS-CoV-2 variants.

The S protein contains a receptor-binding domain (RBD) that binds to human angiotensin-converting enzyme 2 (hACE2) as a receptor to facilitate membrane fusion and cell entry^1^. Ninety percent of the neutralizing antibodies (nAbs) elicited in COVID-19 patients recognize the RBD; therefore, targeting the RBD is an essential vaccine development strategy^2,3^. In late November of 2021, the omicron (B.1.1.529) variant emerged, which carries numerous S protein substitutions (>30), including at least 15 in the RBD. Since then, various omicron lineages (i.e., BA.2, BA.4, BA.5, BA.2.75, BQ.1.1, XBB, XBB.1.5, XBB.1.16, EG.5.1, and BA.2.86) have emerged with additional S substitutions or deletions. The amino acids changes in the omicron variants lead to reduced reactivity to antibodies in sera and/or plasma from convalescent COVID-19 patients and vaccinated individuals compared to the ancestral strain^4–6^. To enhance the efficacy of authorized mRNA vaccines encoding the full-length S protein against omicron variants, bivalent COVID-19 vaccines containing mRNAs encoding the Wuhan-1 and BA.4/5 spike proteins were authorized as a booster vaccine and have been shown to enhance humoral immune responses against XBB.1.5, which was a dominant variant around the world from March to July, 2023^7–10^.

We previously reported the generation of an mRNA vaccine candidate with HPLC-purified mRNA encoding the SARS-CoV-2 S protein RBD (319–541 aa) (derived from the ancestral Wuhan-1 spike gene) encapsulated in lipid nanoparticles (ancestral-type LNP-mRNA-RBD). This LNP-mRNA-RBD induced humoral and cellular immune responses in mice and macaques and reduced viral burdens in macaques infected with ancestral SARS-CoV-2^11^.

Although RBD-based vaccines have been developed by several groups and these vaccines were effective against multiple SARS-CoV-2 variants^12–18^, their effectiveness against recently circulating variants, such as BA.5 or XBB.1.5, which are antigenically distinct from the ancestral strain, has not been examined. We, therefore, evaluated the adaptive immune responses and protective effects of the ancestral-type LNP-mRNA-RBD against recently circulating variants. In addition, we developed LNP-mRNA-RBD encoding the RBD derived from the BA.4/5 spike gene and elucidated its immunogenicity and protective efficacy against recently circulating variants.

## Results

### Cellular and humoral immunity induced by LNP-mRNA-RBD derived from the ancestral strain

**Fig. 1:**
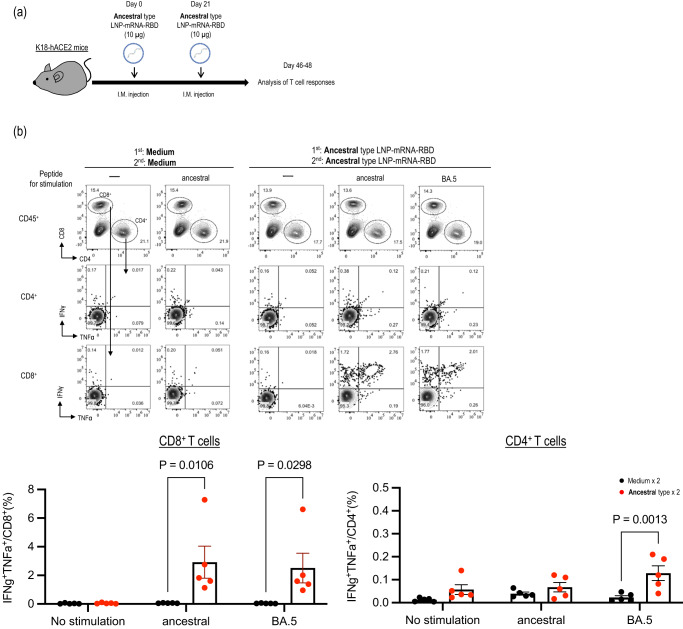
RBD-specific cellular immunity induced in mice immunized with an ancestral-type LNP-mRNA-RBD. **a** Schematic diagram showing the experimental workflow. K18-hACE2 mice were immunized with LNP-mRNA-RBD by intramuscular inoculation, followed by a second booster dose 21 days later. **b** At 3–4 weeks after the second immunization, splenocytes from the immunized mice were re-stimulated with peptide pools designed from ancestral or BA.5 S protein. Top panels: IFN-γ– and TNF-α–secreting cells from spleens were analyzed by flow cytometry after 6 h of stimulation with 1 μg/mL peptide pool. Representative FACS plots are shown for CD4^+^ and CD8^+^ T cells, gated on live CD45^+^ cells. Bottom panels: The frequencies of IFN-γ^+^ TNF-α^+^ cells are represented as the mean ± s.e.m. (*n* = 5/group). Points indicate data from individual mice. Data were analyzed using a two-way ANOVA with Tukey’s multiple comparisons test. Data are from one experiment.

**Fig. 2:**
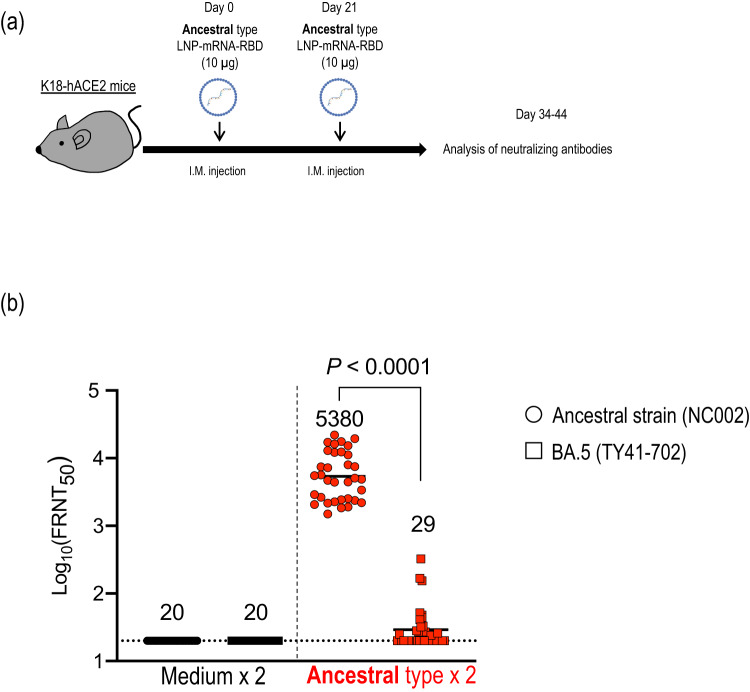
RBD-specific humoral immunity induced in mice immunized with an ancestral-type LNP-mRNA-RBD. **a** Schematic diagram showing the experimental workflow. K18-hACE2 mice were immunized with LNP-mRNA-RBD by intramuscular inoculation, followed by a second booster dose 21 days later (*n* = 30, mock-immunized group; *n* = 34, LNP-mRNA-RBD-immunized group). **b** At 2–3 weeks after the second immunization, serum was collected from the immunized mice. The neutralizing titers (FRNT_50_ values) of the serum samples were determined in Vero E6-TMPRSS2-T2A-ACE2 cells. Each dot represents data from one mouse. The lower limit of detection (value = 20) is indicated by the horizontal dashed line. Samples under the detection limit (<20-fold dilution) were assigned an FRNT_50_ of 20. Geometric mean titers are shown. Data were analyzed with the Wilcoxon matched-pairs signed rank test. Data are from one experiment.

First, we assessed antigen-specific cell-mediated immune responses (Fig. 1a) induced by the LNP-mRNA-RBD derived from an ancestral strain. We found that K18-hACE2 mice (C57BL/6 background)^19^ intramuscularly immunized twice with the ancestral-type LNP-mRNA-RBD dominantly produced S antigen-specific polyfunctional CD8^+^ T cells rather than CD4^+^ T cells after stimulation of peptide pools designed from the ancestral strain. Interestingly, despite 17 mutations in the RBD of omicron BA.5 compared with those in the ancestral strain, the level of induction of antigen-specific T cells was comparable after stimulation with BA.5 peptide pools (Fig. 1b). Next, we determined the 50% focus reduction neutralization titer (FRNT_50_) of serum from immunized mice against the authentic ancestral strain (SARS-CoV-2/UT-NC002-1T/Human/2020/Tokyo: NC002) and BA.5 (hCoV-19/Japan/TY41-702/2022: TY41-702) by using a live-virus neutralization assay. We found that LNP-mRNA-RBD elicited high levels of neutralizing activity against the ancestral strain, with the geometric mean FRNT_50_ (50% neutralization titers) (GMT) value approaching 5380 after two vaccine doses (Fig. 2). However, the neutralizing titers against BA.5 (hCoV-19/Japan/TY41-702/2022: TY41-702) were 186-fold lower than those against the ancestral strain, as expected based on data from studies with human antibodies^8–10^.

### Protection against the ancestral strain and BA.5 by LNP-mRNA-RBD derived from the ancestral strain

**Fig. 3:**
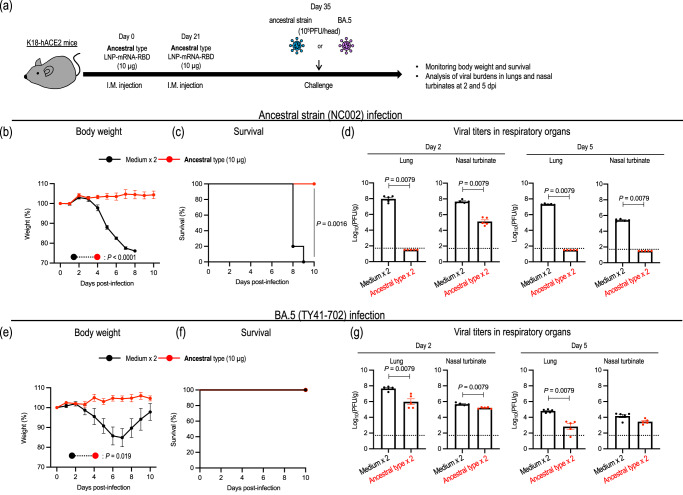
Antigenically matched and distinct variant challenge of mice immunized with an ancestral-type LNP-mRNA-RBD. **a** Schematic diagram showing the experimental workflow. K18-hACE2 mice immunized with LNP-mRNA-RBD twice were challenged with 10^5^ PFU of the ancestral strain (NC002) (**b**–**d**) or BA.5 (TY41-702) (**e**–**g**). **b**, **c**, **e**, **f** Body weights (**b**, **e**) and survival (**c**, **f**) of virus-infected (*n* = 5) and mock-infected (*n* = 5) mice were monitored daily for 10 days after viral infection. Data are the mean percentage ± s.e.m.. of the starting weight. Weight changes were analyzed by using an area under the curve analysis and a two-sided Student’s *t* test. Survival data were analyzed with the log-rank (Mantel–Cox) test. **d**, **g** Five mice per group were euthanized at 2 or 5 dpi for virus titration. Virus titers in the nasal turbinates and lungs were determined using plaque assays. Data are the mean ± s.e.m.; points represent data from individual mice; the lower limit of detection is indicated by the horizontal dashed line. Data were analyzed with the two-tailed Mann–Whitney test. Data are from one experiment.

Since the ancestral-type LNP-mRNA-RBD elicited antigen-specific antibodies, consistent with a previous report^11^, as well as cross-reactive cellular responses in K18-hACE2 mice, we next evaluated the protective effect of the ancestral-type LNP-mRNA-RBD against the ancestral strain or BA.5 strain in mice (Fig. 3).

All mock-immunized mice exhibited severe body weight loss and succumbed to challenge with the ancestral strain (NC002). In contrast, no mice immunized with the ancestral-type LNP-mRNA-RBD lost body weight or died upon challenge with NC002, and the virus titers in their respiratory organs were significantly reduced compared to those of mock-immunized mice (Fig. 3b–d). Interestingly, although no BA.5-infected mice succumbed to their infection due to the lower pathogenicity of this strain compared to the ancestral strain, immunization with the ancestral-type LNP-mRNA-RBD also suppressed body weight loss after BA.5 infection and reduced viral titers in respiratory organs. However, its effectiveness in suppressing viral loads in respiratory organs after infection with omicron variants was attenuated compared to its effectiveness after infection with the antigenically matched virus (NC002) (Fig. 3e–g). These data indicate that LNP-mRNA-RBD based on the ancestral strain protects mice from not only the antigenically matched ancestral strain, but also the antigenically different BA.5 strain. However, its ability to reduce the virus burden in the respiratory organs after infection with the BA.5 strain was limited, suggesting that there is a need to develop a more effective vaccine.

### Cellular and humoral immunity induced by LNP-mRNA-RBD derived from the BA.4/5 strain

**Fig. 4:**
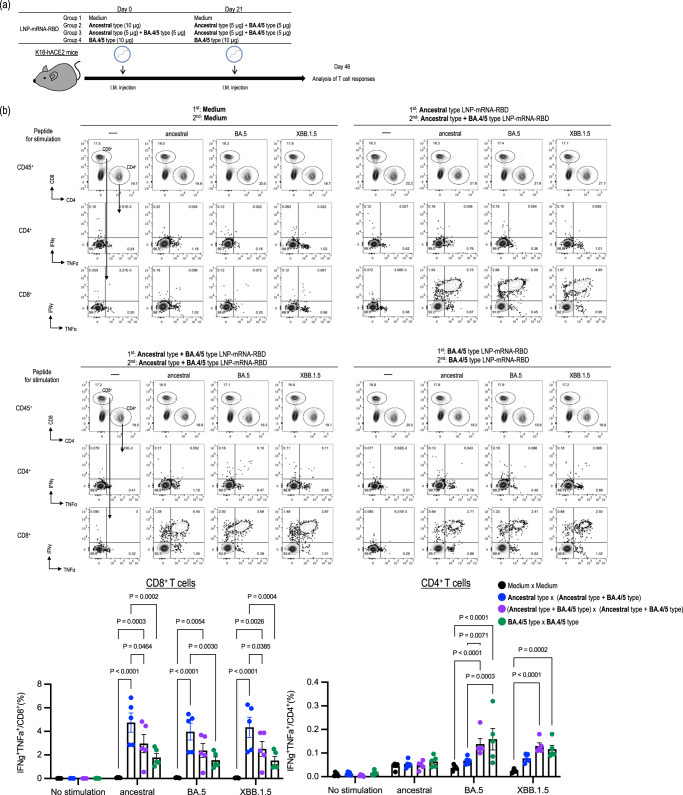
RBD-specific cellular immunity induced in mice immunized with the BA.4/5-type or bivalent LNP-mRNA-RBD. **a** Schematic diagram showing the experimental workflow. K18-hACE2 mice were immunized with LNP-mRNA-RBD derived from the ancestral strain or the BA.4/5 strain by intramuscular inoculation, followed by a second booster dose 21 days later. The details of the groups are described in the table. **b** At 4 weeks after the second immunization, splenocytes from the immunized mice were re-stimulated with peptide pools designed from the ancestral, BA.5, or XBB.1.5S protein. Top panels: IFN-γ– and TNF-α–secreting cells from spleens were analyzed by flow cytometry after 6 h of stimulation with 1 μg/mL peptide pools. Representative FACS plots are shown for CD4^+^ and CD8^+^ T cells, gated on live CD45^+^ cells. Bottom panels: The frequencies of IFN-γ^+^ TNF-α^+^ cells are represented as the mean ± s.e.m. (*n* = 5/group). Points indicate data from individual mice. Data were analyzed by using a two-way ANOVA with Tukey’s multiple comparisons test. Data are from one experiment.

**Fig. 5:**
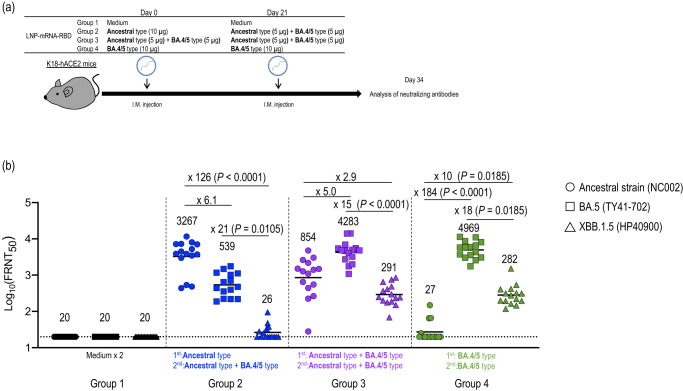
RBD-specific humoral immunity induced in mice immunized with the BA.4/5-type or bivalent LNP-mRNA-RBD. **a** Schematic diagram showing the experimental workflow. K18-hACE2 mice were immunized with LNP-mRNA-RBD derived from the ancestral strain or the BA.4/5 strain by intramuscular inoculation, followed by a second booster dose 21 days later (*n* = 15/group). The details of the groups are described in the table. **b** At 2 weeks after the second immunization, serum was collected from the immunized mice. The neutralizing titers (FRNT_50_ values) of the serum samples were determined in Vero E6-TMPRSS2-T2A-ACE2 cells. Each dot represents data from one mouse. The lower limit of detection (value = 20) is indicated by the horizontal dashed line. Samples under the detection limit (<20-fold dilution) were assigned an FRNT_50_ of 20. Geometric mean titers are shown. Data were analyzed with the Friedman test followed by Dunn’s test. Data are from one experiment.

The newly introduced bivalent mRNA vaccines that express the ancestral strain and omicron S proteins enhance neutralizing activities against omicron variants in humans^20–22^. Therefore, we developed LNP-mRNA-RBD based on the sequence of BA.4/5 and immunized K18-hACE2 mice with different combinations of LNP-mRNA-RBD. Specifically, we assessed the following four groups: a negative control group that received medium twice (Group 1); a group that received the ancestral-type LNP-mRNA-RBD followed by a bivalent vaccine consisting of the ancestral-type and the BA.4/5-type LNP-mRNA-RBD (Group 2); a group that received the bivalent vaccine twice (Group 3); and a group that received the BA.4/5-type LNP-mRNA-RBD twice (Group 4; Figs. 4a and 5a).

First, we examined antigen-specific cell-mediated immune responses (Fig. 4)　induced by LNP-mRNA-RBD derived from a BA.4/5 strain by using K18-hACE2 mice. In each group, the level of induction of antigen-specific IFNγ^+^TNFα^+^ CD8^+^ T cells was comparable when stimulated with the ancestral, BA.5, or XBB.1.5S peptide pools. Interestingly, the ratio of IFNγ^+^TNFα^+^ CD8^+^ T cells from mice immunized with the BA.4/5-type LNP-mRNA-RBD twice was significantly lower than that from mice immunized with the ancestral-type LNP-mRNA-RBD followed by the bivalent vaccine when stimulated with peptide from the ancestral, BA.5, or XBB.1.5 strain (Fig. 4b).

Consistent with immunization with the ancestral-type LNP-mRNA-RBD, the magnitude of the antigen-specific CD4^+^T cell induction was smaller than that of the antigen-specific CD8^+^T cell induction. Of note, the ratios of IFNγ^+^TNFα^+^ CD4^+^ T cells from mice immunized twice with the BA.4/5-type LNP-mRNA-RBD or bivalent LNP-mRNA-RBD were higher than that from mice immunized with the ancestral-type LNP-mRNA-RBD followed by the bivalent vaccine when stimulated with peptide pools from the ancestral, BA.5, or XBB.1.5 strain (Fig. 4b).

Next, we determined the FRNT_50_ of serum against the authentic ancestral strain (NC002), BA.5 (TY41-702), or XBB.1.5 (hCoV-19/USA/MD-HP40900-PIDYSWHNUB/2022: HP40900) by using a live-virus neutralization assay (Fig. 5). We found that serum from mice immunized with the ancestral-type LNP-mRNA-RBD followed by the bivalent vaccine showed a high level of neutralizing activity against the ancestral strain (Fig. 5b group 2, GMT: 3267), but approximately 6.1- and 126-fold reductions against BA.5 (Fig. 5b group 2, GMT: 539), and XBB.1.5, respectively (Fig. 5b group 2, GMT: 26).

We observed a different pattern in mice immunized with the bivalent LNP-mRNA-RBD twice. Immunization with two shots of bivalent vaccine induced high neutralizing titers against BA.5 (Fig. 5b group 3, GMT: 4283), and all immunized mice showed relatively moderate neutralizing activity against both the ancestral strain (Fig. 5b group 3, GMT: 854) and XBB.1.5 (Fig. 5b group 3, GMT: 291).

Mice immunized with BA.4/5-type LNP-mRNA-RBD twice showed high neutralizing titers against BA.5 (Fig. 5b group 4, GMT: 4969); however, the neutralizing titers were 184-fold lower against the ancestral strain (Fig. 5b group 4, GMT: 27) and most serum samples (11 of 15) from mice immunized with the BA.4/5-type LNP-mRNA-RBD twice did not neutralize the ancestral strain (Fig. 5b, group 4). Interestingly, although the neutralizing titers against XBB.1.5 were similar between mice immunized with the bivalent LNP-mRNA-RBD twice (GMT: 291) and those immunized with monovalent BA.4/5-type LNP-mRNA-RBD twice (GMT: 282), the neutralizing activity against the ancestral strain was substantially lower in mice immunized with the BA.4/5-type LNP-mRNA-RBD (GMT: 27) compared to mice immunized with the bivalent LNP-mRNA-RBD (GMT: 291) (Fig. 5b group 3 and group 4). These results suggest that two shots of the bivalent LNP-mRNA-RBD vaccine can induce broadly reactive antibody responses.

### Protection against the ancestral strain and omicron variants by LNP-mRNA-RBD derived from the BA.4/5 strain

**Fig. 6:**
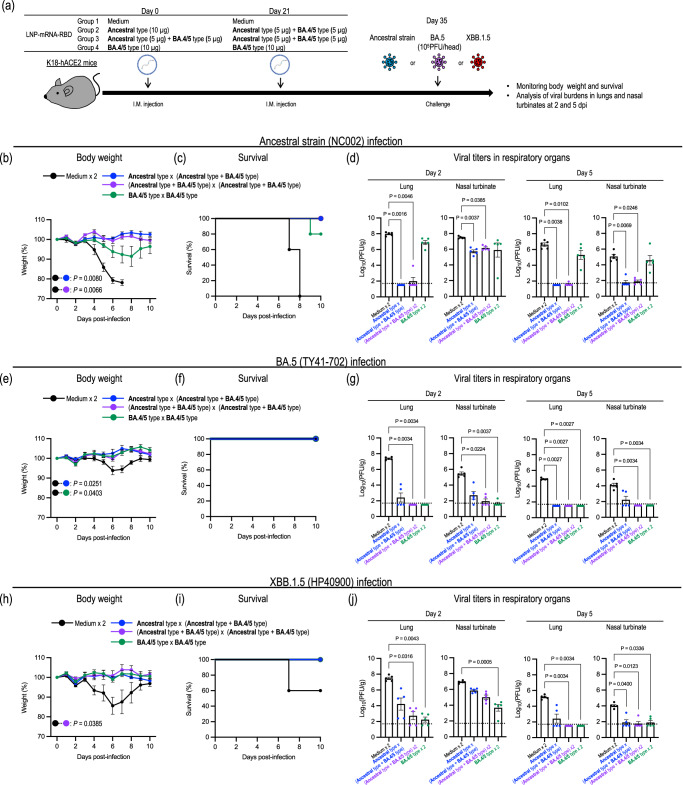
Antigenically matched and distinct variant challenge of mice immunized with the BA.4/5-type or bivalent LNP-mRNA-RBD. **a** Schematic diagram showing the experimental workflow. K18-hACE2 mice immunized with LNP-mRNA-RBD derived from the ancestral strain or the BA.4/5 strain were challenged with 10^5^ PFU of the ancestral strain (NC002) (**b**–**d**), BA.5 (TY41-702) (**e**–**g**), or XBB.1.5 (HP40900) (**h**–**j**). The details of the groups are described in the table. **b**, **c**, **e**, **f**, **h**, **i** Body weights (**b**, **e**, **h**) and survival (**c**, **f**, **i**) of virus-infected (*n* = 5) and mock-infected (*n* = 5) mice were monitored daily for 10 days after viral challenge. Data are the mean percentage ± s.e.m. of the starting weight. Weight changes were analyzed by using an area under the curve analysis and a one-way ANOVA with Tukey’s multiple comparisons test (**e**) or the Kruskal–Wallis test with Dunn’s multiple comparisons (**b**, **h**). Survival data were analyzed with the log-rank (Mantel–Cox) test. **d**, **g**, **j** Five mice per group were euthanized at 2 or 5 dpi for virus titration. Virus titers in the nasal turbinates and lungs were determined using plaque assays. Data are the mean ± s.e.m.; points represent data from individual mice; the lower limit of detection is indicated by the horizontal dashed line. Data were analyzed with the Kruskal–Wallis test with Dunn’s multiple comparisons. Data are from one experiment.

To assess the protective effect of the BA.4/5-based LNP-mRNA-RBD vaccine, we performed challenge studies in K18-hACE2 mice (Fig. 6a). Cohorts of female K18-hACE2 mice were intramuscularly immunized with (1) the ancestral-type LNP-mRNA-RBD followed by the bivalent LNP-mRNA-RBD, (2) the bivalent LNP-mRNA-RBD twice, (3) the BA.4/5-type LNP-mRNA-RBD, or (4) medium twice. Although the degree of body weight change was attenuated in BA.5- or XBB.1.5-infected mice compared to mice infected with the ancestral strain in the mock-immunized groups due to the lower pathogenicity of these strains, body weight loss was observed within 6 days of infection with the ancestral, BA.5, or XBB.1.5 strain in mock-immunized mice. Importantly, the weight loss and lethality caused by these viruses was prevented in mice immunized with the ancestral-type LNP-mRNA-RBD followed by bivalent LNP-mRNA-RBD or the bivalent vaccine twice (Fig. 6b, c, e, f, h, i). The BA.4/5-type LNP-mRNA-RBD prevented mice from losing body weight after BA.5 or XBB.1.5 challenge (Fig. 6e, f, h, i); however, upon ancestral strain challenge, mice immunized only with the BA.4/5-type vaccine showed body weight loss and one of five succumbed to the infection with the ancestral strain (Fig. 6b, c).

We also examined the virus titers in the lungs and nasal turbinates after challenge with the ancestral, BA.5, or XBB.1.5 strain. After challenge with the ancestral strain, high levels of virus were detected in the lungs and nasal turbinates of the mock-immunized mice. Immunization with the ancestral-type vaccine followed by the bivalent vaccine or two shots of bivalent vaccine conferred significant protection in both the lungs and nasal turbinates with significant reductions in viral titers at 2 and 5 dpi, compared to those titers in mock-immunized mice (mean reductions in viral titers of 5.0–6.5 log_10_ PFU g^−1^ in the lungs; mean reductions in viral titers of 1.3–3.3 log_10_ PFU g^−1^ in the nasal turbinates) (Fig. 6d). In contrast, the effect of the BA.4/5-type vaccine was limited, as demonstrated by only a slight reduction in virus titers in the respiratory organs (Fig. 6d). After BA.5 challenge, all vaccinations led to remarkable (10^1.9^- to 10^5.9^-fold) reductions in virus titers relative to mock-immunization, although the difference in virus titers between mock-immunized mice and mice immunized with the ancestral-type vaccine followed by the bivalent vaccine did not reach statistical significance (Fig. 6g). A similar trend was observed after XBB.1.5 challenge; immunization with two shots of the bivalent vaccine or the BA.4/5-type vaccine conferred significant protection in the respiratory organs (Fig. 6j). Although the degree of reduction was limited, the ancestral-type vaccine followed by the bivalent vaccine also suppressed virus infection in the lungs (mean reductions in viral titers of 3.3 and 2.7 log_10_ PFU g^−1^ at 2 and 5 dpi, respectively) and nasal turbinates (mean reductions in viral titers of 1.1 and 2.1 log_10_ PFU g^−1^ at 2 and 5 dpi, respectively).

Overall, these results suggest that two shots of the bivalent LNP-mRNA-RBD vaccine can induce cross-protection against a broad spectrum of SARS-CoV-2 strains.

## Discussion

Here, we showed that the ancestral-type LNP-mRNA-RBD inhibited body weight loss after challenge with the ancestral strain or BA.5, although suppression of viral burdens in the respiratory organs after BA.5 infection was attenuated compared with that after infection with the ancestral strain (Figs. 1–3).

To improve the breadth of elicited adaptive immunity, we developed a BA.4/5-type LNP-mRNA-RBD encoding the RBD of the BA.4/5S protein and investigated the efficacy of this vaccine against the ancestral strain, BA.5, and XBB.1.5. Two shots of the bivalent LNP-mRNA-RBD or the monovalent BA.4/5-type LNP-mRNA-RBD triggered strong cellular immune responses and resulted in high and moderate neutralizing titers against BA.5 and XBB.1.5, respectively (Figs. 4 and 5). Nonetheless, compared to the neutralizing activity against BA.5, the activity against the ancestral strain was substantially attenuated (184-fold) in mice vaccinated with the monovalent BA.4/5-type LNP-mRNA-RBD, whereas a modest decrease (5-fold) was observed in mice immunized with the bivalent LNP-mRNA-RBD (Fig. 5). Our challenge study demonstrated that two shots of the bivalent LNP-mRNA-RBD or the monovalent BA.4/5-type LNP-mRNA-RBD reduced the viral burden in the respiratory organs after BA.5 or XBB.1.5 challenge. While the bivalent LNP-mRNA-RBD also protected mice from the lethal challenge of the ancestral strain, the efficacy of the monovalent BA.4/5-type LNP-mRNA-RBD against the ancestral strain was attenuated (Fig. 6). These results suggest that a bivalent vaccine containing the antigens of the ancestral and currently circulating strains could increase the breadth of protection against omicron variants.

Regarding antibody induction, immunization with the bivalent vaccine after immunization with the ancestral-type LNP-mRNA-RBD improved the neutralizing titers against BA.5 (GMT:539 in Fig. 5b) relative to immunization with two shots of the ancestral-type vaccine (GMT:29 in Fig. 2b), but 60% of the tested mice (9 of 15 immunized mice) showed FRNT_50_ titers that were below the detection limit (<20) against XBB.1.5. Although prime/boost immunization with the bivalent LNP-mRNA-RBD induced neutralizing antibodies against both BA.5 and XBB.1.5 in all immunized mice, the antibody titers against XBB.1.5 were markedly lower. This reduction may be caused by the additional substitutions in the XBB.1.5 RBD (G339H, R346T, L368I, V445P, G446S, N460K, F486P, and F490S) compared with the BA.4/5 RBD, given that previous studies have demonstrated that mutations introduced in XBB.1.5 contribute to escape from neutralizing antibody responses in humans^8–10^. Recently, an mRNA vaccine encoding the full-length XBB.1.5 spike protein has been made available and a booster shot with this vaccine has been shown to enhance the neutralizing antibodies in the serum of vaccinees against XBB.1.5 and recently emerging strains, including XBB.1.16, EG.5.1, and BA.2.86^23^. Considering that the antigenicity of recently circulating strains differs slightly from that of XBB.1.5 but is closer than that of the ancestral strain or BA.5^24,25^, the development of an mRNA vaccine with LNP-mRNA-RBD based on the sequence of XBB.1.5 may improve immune responses and protection against recently circulating strains compared to an ancestral-type or BA.4/5-type LNP-mRNA-RBD.

Previous studies have shown that T cell responses play a critical role in virus clearance post-challenge with SARS-CoV and SARS-CoV-2, and that T cell vaccination in the absence of neutralizing antibodies can protect mice from SARS-CoV-2 infection^26–28^. Here, although serum from mice immunized with an ancestral-type LNP-mRNA-RBD or from mice immunized with an ancestral-type vaccine followed by a bivalent vaccine had remarkably low neutralizing activities against BA.5 or XBB.1.5, respectively, these immunized mice were protected from clinical signs of illness and had reduced viral burdens in their respiratory organs after BA.5 or XBB.1.5 challenge, respectively. Given that these immunized mice produced antigen-specific cellular responses, these results suggest that cellular immune responses, as well as neutralizing activity induced by LNP-mRNA-RBD, contribute to antigenically different virus clearance from K18-hACE2 mice.

Interestingly, the induction of IFNγ^+^TNFα^+^ CD8^+^ T cells was significantly reduced in mice immunized twice with the BA.4/5-type LNP-mRNA-RBD compared with mice immunized with the ancestral-type LNP-mRNA-RBD followed by the bivalent vaccine. Conversely, the opposite trend was observed with respect to the induction of IFNγ^+^TNFα^+^ CD4^+^ T cells. T cell responses after vaccination are induced by interactions among MHC class I/II, antigenic SARS-CoV-2 peptides and T-cell receptors^29^. Although further studies are required, differences between the ancestral and BA.4/5 RBD may account for the pattern of antigen-specific T cell responses induced by the LNP-mRNA-RBD developed in this study, as a previous study demonstrated that some mutations introduced in the BA.4/5 RBD may generate or inactivate MHC-I/II-restricted epitopes^30^.

We note several limitations in our study: (1) It is unclear how long the adaptive immunity induced by LNP-mRNA-RBD lasts. Further evaluation of the persistence of adaptive immunity in humans necessitates clinical data^31^; (2) We evaluated the efficacy of LNP-mRNA-RBD in healthy young mice. Previous studies have shown that the efficacy of mRNA-1273 or bNT162b2 is affected by the age and underlying health conditions of vaccinees^32–35^. Therefore, additional studies should be undertaken to assess the extent to which host comorbidities affect the effectiveness of LNP-mRNA-RBD; and (3) Much of the world’s population has now been vaccinated or infected with SARS-CoV-2. Given that imprinting of humoral immunity reduces the diversity of neutralizing antibodies against SARS-CoV-2^36^, it is important to evaluate the impact of pre-immunity on the efficacy of LNP-mRNA-RBD.

Overall, our studies provide evidence that bivalent LNP-mRNA-RBD or monovalent BA.4/5-type LNP-mRNA-RBD can induce protective immune responses that extend to antigenically distinct XBB.1.5 viruses even though the neutralizing activity against XBB.1.5 is somewhat attenuated. Since XBB.1-descendent lineages are circulating dominantly in the world, the development of mRNA vaccine with LNP-mRNA-RBD based on the sequence of an XBB.1 descendent should be considered to improve immune responses and protective efficacy against recently circulating strains.

## Methods

### Cells

VeroE6/TMPRSS2 (JCRB 1819) cells were propagated in the presence of 1 mg/ml geneticin (G418; Invivogen) and 5 μg/ml plasmocin prophylactic (Invivogen) in Dulbecco’s modified Eagle’s medium (DMEM) containing 10% Fetal Calf Serum (FCS). Vero E6-TMPRSS2-T2A-ACE2 cells (provided by Dr. Barney Graham, NIAID Vaccine Research Center) were cultured in DMEM supplemented with 10% FCS, 100 U/mL penicillin–streptomycin, and 10 μg/mL puromycin. VeroE6/TMPRSS2 and Vero E6-TMPRSS2-T2A-ACE2 cells were maintained at 37 °C with 5% CO_2_. Expi293 cells (Thermo Fisher) were maintained in Expi293 expression medium (Thermo Fisher) at 37 °C under 8% CO_2_. The cells were regularly tested for mycoplasma contamination by using PCR and confirmed to be mycoplasma-free.

### Viruses

hCoV-19/USA/MD-HP40900-PIDYSWHNUB/2022 (omicron XBB.1.5: HP40900)^8^, hCoV-19/Japan/TY41-702/2022 (omicron BA.5; TY41-702)^37^ and SARS-CoV-2/UT-NC002-1T/Human/2020/Tokyo (ancestral; NC002) were propagated in VeroE6/TMPRSS2 cells in VP-SFM (Thermo Fisher Scientific). All experiments with SARS-CoV-2 were performed in enhanced biosafety level 3 (BSL3) containment laboratories at the University of Tokyo and the National Institute of Infectious Diseases, Japan, which are approved for such use by the Ministry of Agriculture, Forestry, and Fisheries, Japan.

### mRNA vaccine

T7 RNA polymerase-mediated transcription in vitro was used to synthesize the mRNA from a linearized DNA template, in which the open-reading frame of the RBD was flanked by 5’ and 3’ untranslated regions and a poly-A tail. Messenger RNA for the RBD (HPLC) was purified by reversed-phase chromatography and then encapsulated into lipid nanoparticles (LNPs) composed of ionizable lipid, phospholipid, cholesterol, and PEG-lipid.

### Animal experiments and approvals

Animal studies were carried out in accordance with the recommendations in the Guide for the Care and Use of Laboratory Animals of the National Institutes of Health. The protocol was approved by the Institutional Animal Care and Use Committee at the Animal Experiment Committee of the Institute of Medical Science, the University of Tokyo (approval number: PA19-72). All animals were housed under specific pathogen-free conditions in a temperature control environment with a 12 h: 12 h light: dark cycle, with 50% humidity and ad libitum access to water and standard laboratory chow. Virus inoculations were performed under anesthesia with isoflurane, and all efforts were made to minimize animal suffering. In vivo studies were not blinded, and animals were randomly assigned to immunization/infection groups. No sample-size calculations were performed to power each study. Instead, sample sizes were determined based on prior in vivo virus challenge experiments.

### Immunization and challenge

Hemizygous K18-hACE2 C57BL/6 J mice (strain B6.Cg-Tg(K18-ACE2)2Prlmn/J) were obtained from the Jackson Laboratory. For the immunization and protection studies, 6–8 week-old female K18-hACE2 mice were anesthetized with isoflurane and intramuscularly mock-immunized or immunized with LNP-mRNA-RBD twice with a 3-week interval between immunizations; 2–3 weeks after the second immunization, the mice were intranasally challenged with 10^5^ PFU of SARS-CoV-2. Baseline body weights were measured before infection. Body weight and survival were monitored daily for 10 days. Mice with more than 25% body weight loss were euthanized with deep anesthesia by isoflurane and cervical dislocation. For examination of viral burdens in respiratory organs, mice were euthanized with deep anesthesia by isoflurane and cervical dislocation on days 2 and 5 post-infection, and their lungs and nasal turbinates were collected and examined for virus titers by using plaque assays.

### Focus reduction neutralization test

Neutralization activities of serum were determined by using a focus reduction neutralization test as previously described^38^. The samples were first incubated at 56 °C for 1 h. Then, the treated serum samples were serially diluted five-fold with DMEM containing 2% FCS in 96-well plates and mixed with 100–500 FFU of virus/well, followed by incubation at 37 °C for 1 h. The serum-virus mixture was inoculated onto Vero E6-TMPRSS2-T2A-ACE2 cells in 96-well plates in duplicate and incubated for 1 h at 37 °C. After a 1-h incubation at 37 °C, 100 μl of 1.5% Methyl Cellulose 400 (FUJIFILM Wako Pure Chemical Corporation) in culture medium was then added to each well. The cells were incubated for 14–16 h at 37 °C and then fixed with formalin.

After the formalin was removed, the cells were immunostained with a rabbit monoclonal antibody against SARS-CoV-2 nucleoprotein (sino biological Inc., dilution: 1:10,000, Cat #: 40143-R001) followed by a horseradish peroxidase-labeled goat anti-rabbit immunoglobulin (Jackson ImmunoResearch Laboratories Inc., dilution: 1:2,000, Cat #: 111-035-003). The infected cells were stained with TrueBlue Substrate (SeraCare Life Sciences) and then washed with distilled water. After cell drying, the focus numbers were quantified by using an ImmunoSpot S6 Analyzer, ImmunoCapture software, and BioSpot software (Cellular Technology). The results are expressed as the 50% focus reduction neutralization titer (FRNT_50_). The FRNT_50_ values were calculated by using GraphPad Prism (GraphPad Software). Samples under the detection limit (<20-fold dilution) were assigned an FRNT_50_ of 20.

### Intracellular cytokine staining

To harvest single cells from immunized mouse spleen, spleens were minced to yield 1–2 mm pieces and incubated with Hanks’ Balanced Salt Solution (HBSS) containing collagenase D for at least 15 min at 37 °C. After treatment with red blood cell lysis buffer on ice, the cells were resuspended in RPMI 1640 with 10% heat-inactivated FCS, 100 units/mL penicillin, and 100 μg/mL streptomycin. One million cells from the spleens of immunized mice were stimulated for 6 h with or without 1 μg/mL SARS-CoV-2 peptide designed from the ancestral strain (peptides and elephants, Hennigsdorf, Germany), omicron BA.5 (Genaxxon Bioscience, Biberach, Germany) in the presence of GolgiStop (BD Biosciences, Sparks, MD, USA), or XBB.1.5 (peptides and elephants, Hennigsdorf, Germany) in a U-bottom plate at 37 °C under 5% CO_2_ in RPMI 1640 with 10% heat-inactivated FCS. After incubation, the cells were incubated with Live/dead fixable aqua (Thermo Fisher Scientific, Waltham, MA, USA), anti-CD16/32 (BD Biosciences, clone 93, dilution: 1:200 ~ 1:250, Cat #: 553142) Ab, and antibodies specific to CD45 (Biolegend, clone 30-F11, dilution: 1:100, Cat #: 103128), CD4 (Biolegend, clone GK1.5, dilution: 1:100, Cat #: 100434), and CD8a (Biolegend, clone 53-6.7, dilution: 1:100, Cat #: 100723). Following fixation and permeabilization with Cytofix/Cytoperm from the Fixation/Permeabilization Solution Kit (BD Biosciences), cells were stained with antibodies specific to TNF-α (Biolegend, clone MP6-XT22, dilution: 1:100, Cat #: 506314) and IFN-γ (Biolegend, clone XMG1.2, dilution: 1:50, Cat #: 505808).

Data were acquired with CytoFLEX S (Beckman Coulter Inc., Brea, CA, USA) and data analysis was performed using FlowJo software (FlowJo, Ashland, OR, USA).

### Statistics

GraphPad Prism 9.3.1 software was used to perform all statistical analysis. No statistical methods were used to predetermine sample size. A *P* value less than 0.05 was considered statistically significant.

### Reagent availability

LNP-mRNA-RBD was prepared by Daiichi Sankyo Co., Ltd. and used for the current research only. Other materials are available from the authors or from commercially available sources.

### Reporting summary

Further information on research design is available in the Nature Research Reporting Summary linked to this article.

### Supplementary information

Reporting summary

## Data Availability

All data supporting the findings of this study are available in the paper. There are no restrictions to obtaining access to the primary data.
