# Supplementary material for: An mRNA vaccine encoding the SARS-CoV-2 receptor-binding domain protects mice from various Omicron variants
Source: NPJ Vaccines. 2024 Jan 2;9:4. doi: 10.1038/s41541-023-00800-0 (PMC10761957; doi:10.1038/s41541-023-00800-0)
Supplement: Supplementary file 1 — Reporting summary [file 41541_2023_800_MOESM1_ESM.pdf]

Corresponding author(s): Yoshihiro Kawaoka

Last updated by author(s): 2023/12/2

Reporting Summary

Nature Portfolio wishes to improve the reproducibility of the work that we publish. This form provides structure for consistency and transparency in reporting. For further information on Nature Portfolio policies, see our Editorial Policies and the Editorial Policy Checklist.

Statistics

For all statistical analyses, confirm that the following items are present in the figure legend, table legend, main text, or Methods section.

n/a	Confirmed	
☐	☒	The exact sample size (*n*) for each experimental group/condition, given as a discrete number and unit of measurement
☐	☒	A statement on whether measurements were taken from distinct samples or whether the same sample was measured repeatedly
☐	☒	The statistical test(s) used AND whether they are one- or two-sided<br>*Only common tests should be described solely by name; describe more complex techniques in the Methods section.*
☒	☐	A description of all covariates tested
☐	☒	A description of any assumptions or corrections, such as tests of normality and adjustment for multiple comparisons
☐	☒	A full description of the statistical parameters including central tendency (e.g. means) or other basic estimates (e.g. regression coefficient) AND variation (e.g. standard deviation) or associated estimates of uncertainty (e.g. confidence intervals)
☐	☒	For null hypothesis testing, the test statistic (e.g. *F*, *t*, *r*) with confidence intervals, effect sizes, degrees of freedom and *P* value noted<br>*Give P values as exact values whenever suitable.*
☒	☐	For Bayesian analysis, information on the choice of priors and Markov chain Monte Carlo settings
☒	☐	For hierarchical and complex designs, identification of the appropriate level for tests and full reporting of outcomes
☒	☐	Estimates of effect sizes (e.g. Cohen's *d*, Pearson's *r*), indicating how they were calculated

Our web collection on statistics for biologists contains articles on many of the points above.

Software and code

Policy information about availability of computer code

Data collection	CytoFLEX S (Beckman Coulter Inc)<br>ImmunoSpot S6 Analyzer (Cellular Technology)
Data analysis	FlowJo software (FlowJo)<br>ImmunoCapture software (Cellular Technology)<br>BioSpot software (Cellular Technology)<br>GraphPad Prism software 9.3.1 was used for the statical analysis.

For manuscripts utilizing custom algorithms or software that are central to the research but not yet described in published literature, software must be made available to editors and reviewers. We strongly encourage code deposition in a community repository (e.g. GitHub). See the Nature Portfolio guidelines for submitting code & software for further information.

Data

Policy information about availability of data

All manuscripts must include a data availability statement. This statement should provide the following information, where applicable:

- Accession codes, unique identifiers, or web links for publicly available datasets
- A description of any restrictions on data availability
- For clinical datasets or third party data, please ensure that the statement adheres to our policy

All data supporting the findings of this study are available in the paper. There are no restrictions to obtaining access to the primary data.

Research involving human participants, their data, or biological material

Policy information about studies with human participants or human data. See also policy information about sex, gender (identity/presentation), and sexual orientation and race, ethnicity and racism.

Reporting on sex and gender	N/A
Reporting on race, ethnicity, or other socially relevant groupings	N/A
Population characteristics	N/A
Recruitment	N/A
Ethics oversight	N/A

Note that full information on the approval of the study protocol must also be provided in the manuscript.

Field-specific reporting

Please select the one below that is the best fit for your research. If you are not sure, read the appropriate sections before making your selection.

☒ Life sciences ☐ Behavioural & social sciences ☐ Ecological, evolutionary & environmental sciences

For a reference copy of the document with all sections, see nature.com/documents/nr-reporting-summary-flat.pdf

Life sciences study design

All studies must disclose on these points even when the disclosure is negative.

Sample size	No sample-size calculations were performed. No statistical method was used to determine sample size. Mouse experiments were performed with at least n = 5 per group. All sample sizes were chosen based on standard practices in the field.
Data exclusions	No data exclusions.
Replication	All experiments with multiple biological replicates are indicated in the figure legends.
Randomization	No method of randomization was used to determine how the animals were allocated to the experimental groups and processed in this study. However, covariates including sex and age were identical in groups.
Blinding	No blinding was carried out due to the limited number of staff available to conduct these studies.

Reporting for specific materials, systems and methods

We require information from authors about some types of materials, experimental systems and methods used in many studies. Here, indicate whether each material, system or method listed is relevant to your study. If you are not sure if a list item applies to your research, read the appropriate section before selecting a response.

Materials & experimental systems

n/a	Involved in the study
☐	☒ Antibodies
☐	☒ Eukaryotic cell lines
☒	☐ Palaeontology and archaeology
☐	☒ Animals and other organisms
☒	☐ Clinical data
☒	☐ Dual use research of concern
☒	☐ Plants

Methods

n/a	Involved in the study
☒	☐ ChIP-seq
☐	☒ Flow cytometry
☒	☐ MRI-based neuroimaging

Antibodies

Antibodies used	a rabbit monoclonal antibody against SARS-CoV-2 nucleoprotein(sino biological Inc.,#: 40143-R001)<br>a horseradish peroxidase-labeled goat anti-rabbit immunoglobulin (Jackson ImmunoResearch Laboratories Inc. #: 111-035-003)<br>Purified Rat Anti-Mouse CD16/CD32 (BD #553142)<br>Alexa Fluor® 700 anti-mouse CD45 Antibody (Biolegend #103128)<br>PerCP/Cyanine5.5 anti-mouse CD4 Antibody (Biolegend #100434)<br>Alexa Fluor® 488 anti-mouse CD8a Antibody (Biolegend #100723)<br>Alexa Fluor® 647 anti-mouse TNF-α Antibody (Biolegend #506314)<br>PE anti-mouse IFN-γ Antibody (Biolegend #505808)
Validation	a rabbit monoclonal antibody against SARS-CoV-2 nucleoprotein: https://www.sinobiological.com/antibodies/cov-nucleocapsid-40143-r001<br>a horseradish peroxidase-labeled goat anti-rabbit immunoglobulin: https://www.jacksonimmuno.com/catalog/products/111-035-003<br>Purified Rat Anti-Mouse CD16/CD32: https://www.bdbiosciences.com/ja-jp/products/reagents/flow-cytometry-reagents/research-reagents/single-color-antibodies-ruo/purified-rat-anti-mouse-cd16-cd32-mouse-bd-fc-block.553142<br>Alexa Fluor® 700 anti-mouse CD45 Antibody: https://www.biolegend.com/ja-jp/soluble-mhc/alexa-fluor-700-anti-mouse-cd45-antibody-3407?GroupID=BLG6833<br>PerCP/Cyanine5.5 anti-mouse CD4 Antibody: https://www.biolegend.com/ja-jp/products/percp-cyanine5-5-anti-mouse-cd4-antibody-4220?GroupID=BLG4745<br>Alexa Fluor® 488 anti-mouse CD8a Antibody: https://www.biolegend.com/ja-jp/cell-health/alexa-fluor-488-anti-mouse-cd8a-antibody-2698<br>Alexa Fluor® 647 anti-mouse TNF-α Antibody: https://www.biolegend.com/ja-jp/products/alexa-fluor-647-anti-mouse-tnf-alpha-antibody-2724<br>PE anti-mouse IFN-γ Antibody: https://www.biolegend.com/ja-jp/products/pe-anti-mouse-ifn-gamma-antibody-997?GroupID=GROUP24

Eukaryotic cell lines

Policy information about cell lines and Sex and Gender in Research

Cell line source(s)	VeroE6-TMPRSS2 cells (available at Japanese Collection of Research Bioresource Cell Bank, JCRB 1819), VeroE6-TMPRSS2-T2A-ACE2, VRC-NIH (available at Bei Resources, NR-54970).
Authentication	VeroE6-TMPRSS2 and VeroE6-TMPRSS2-T2A-ACE2 cells were assumed to be authentic by the cell bank or manufactures. No further authorization was performed by the authors.
Mycoplasma contamination	All cell lines were tested monthly in our laboratory and were negative each time for mycoplasma contamination.
Commonly misidentified lines (See ICLAC register)	No commonly misidentified lines were used in this study.

Animals and other research organisms

Policy information about studies involving animals; ARRIVE guidelines recommended for reporting animal research, and Sex and Gender in Research

Laboratory animals	Hemizygous K18-hACE2 C57BL/6J mice (strain 2B6.Cg-Tg(K18-ACE2)2Prlmn/J, 6–8 week-old) were obtained from the Jackson Laboratory.
Wild animals	No wild animals were used in this study.
Reporting on sex	Female hemizygous K18-hACE2 C57BL/6J mice (strain 2B6.Cg-Tg(K18-ACE2)2Prlmn/J) were used, due to the limited availability.
Field-collected samples	This study did not involve sample collection from the field.
Ethics oversight	Animal studies were carried out in accordance with the recommendations in the Guide for the Care and Use of Laboratory Animals of

Ethics oversight	the National Institutes of Health. The protocol was approved by the Institutional Animal Care and Use Committee at the Animal Experiment Committee of the Institute of Medical Science, the University of Tokyo (Approved number: PA19-72).

Note that full information on the approval of the study protocol must also be provided in the manuscript.

Plants

Seed stocks	N/A
Novel plant genotypes	N/A
Authentication	N/A

Flow Cytometry

Plots

Confirm that:

- [x] The axis labels state the marker and fluorochrome used (e.g. CD4-FITC).
- [x] The axis scales are clearly visible. Include numbers along axes only for bottom left plot of group (a 'group' is an analysis of identical markers).
- [x] All plots are contour plots with outliers or pseudocolor plots.
- [x] A numerical value for number of cells or percentage (with statistics) is provided.

Methodology

Sample preparation	To harvest single cells from immunized mouse spleen, spleens were minced to yield 1–2 mm pieces and incubated with HBSS containing collagenase D for at least 15 min at 37 °C. After treatment with red blood cell lysis buffer, cells were resuspended in RPMI 1640 with 10% FCS, 100 units/mL penicillin, and 100 µg/mL streptomycin. After incubation of stimulated cells, the cells were incubated with Live/dead fixable aqua (Thermo Fisher Scientific), anti-CD16/32 (93) Ab, and antibodies specific to CD45 (30-F11), CD4 (GK1.5), and CD8a (53–6.7). Following fixation and permeabilization with Cytofix/Cytoperm from the Fixation/Permeabilization Solution Kit (BD Biosciences), the cells were stained with antibodies specific to TNF-α (MP6-XT22) and IFN-γ (XMG1.2) (Biolegend).
Instrument	CytoFLEX S (Beckman Coulter Inc)
Software	FlowJo software v10.8.2 (FlowJo LLC)
Cell population abundance	No cell population sorting carried out.
Gating strategy	IFN-γ+ TNF-α+ CD4+ or CD8+ T cells are pre-gated on size, singlets, dead cell stain−, CD45+, and separated by CD4+ or CD8+. Then, the cells which were double-positive with TNF-α and IFN-γ, were gated.

- [ ] Tick this box to confirm that a figure exemplifying the gating strategy is provided in the Supplementary Information.
